# The Role of Brain-Derived Neurotrophic Factor in the Pathophysiology of Psychiatric and Neurological Disorders

**DOI:** 10.26502/jppd.2572-519X0024

**Published:** 2017-07-24

**Authors:** Mariana Angoa-Pérez, John H. Anneken, Donald M. Kuhn

**Affiliations:** 1 Research & Development Service, John D. Dingell VA Medical Center, Detroit, MI 48201, USA; 2 Department of Psychiatry & Behavioral Neurosciences, Wayne State University School of Medicine, Detroit, MI 48201, USA

**Keywords:** BDNF, TrkB, p75NTR, Depression, Alzheimer’s, Parkinson’s, Schizophrenia

## Abstract

Brain-derived neurotrophic factor (BDNF) is a neurotrophin highly expressed in the brain with a potent influence on several aspects of neuronal function. Since its discovery in the early 1980s, BDNF has prompted a great interest in better understanding its physiological role and has been established as the main central neurotrophic factor. BDNF is initially synthesized as a precursor, pro-BDNF, which is then cleaved to form mature BDNF (m-BDNF). A regulated balance between pro-BDNF and m-BDNF is crucial for physiological as well as pathological conditions. The diverse effects of BDNF are mediated through the p75 NT receptor (p75NTR), which binds to its precursor form, and the tropomyosin receptor kinase B (TrkB), which binds to its mature form. Activation of TrkB and p75NTR may produce opposite outcomes in that TrkB receptors have a well-defined trophic role and their activation is proposed to mediate neuronal survival, whereas p75NTR may promote apoptosis. BDNF is highly expressed in limbic structures and cerebral cortex, making it a crucial factor in the regulation of learning and memory, affective behaviors and reward processes. Abnormal BDNF signaling has been proposed to have a crucial role in the course and development of numerous psychiatric and neurological disorders. Moreover, psychotropic drugs used to treat some of these conditions are known to activate BDNF signaling. The present review gives an overview of the involvement of BDNF in the pathology of psychiatric and neurological disorders, compiling what is known from human and animal studies.

## 1. Introduction

Brain-derived neurotrophic factor (BDNF) is a neurotrophin highly expressed in the brain with important roles in neuronal survival, differentiation, morphogenesis and synaptic plasticity [1-3]. As with all neurotrophins, BDNF is initially synthesized as a precursor, pro-BDNF, which is then cleaved either inside the cells or after its secretion to form mature BDNF (m-BDNF) [2]. A particularity not shared with other neurotrophins is that BDNF is secreted through constitutive (both in non-neuronal and neuronal cells) as well as regulated pathways (exclusively in neurons) [2, 4]. A controlled balance between pro-BDNF and m-BDNF is crucial for physiological and also pathological conditions [4].

The diverse effects of BDNF are mediated through the p75 NT receptor (p75NTR), which binds the precursor form, and the tropomyosin receptor kinase B (TrkB), which binds the mature form [2]. BDNF binds to TrkB receptor with high affinity and to p75NTR, with low affinity [5]. While TrkB is broadly expressed in both the developing and adult brain [1], p75NTR expression is downregulated in the adult brain [6]. However, p75NTR expression in damaged or diseased conditions is rapidly induced, which has been associated with neuronal cell death [6]. Activation of TrkB and p75NTR may produce complex and sometimes opposite outcomes. TrkB receptors have a well-defined trophic role and their activation is proposed to mediate neuronal survival, spine formation and maturation [7], whereas p75NTR may promote a variety of functions ranging from trophism to apoptosis [6]. To better illustrate the contrasting role of these receptors, activation of TrkB in the hippocampus contributes to long-term potentiation whereas activation of p75NTR in the same brain structure enhances long-term depression [7]. Similarly, TrkB and p75NTR have been reported to play opposing roles in fear acquisition and anxiety in mice [7].

BDNF is highly expressed in limbic structures and cerebral cortex, making it a crucial factor in the regulation of learning and memory, affective behaviors and reward processes [8]. Human platelets also contain a large amount of the BDNF in blood but the significance of this is not clear. It is known that BDNF can cross the blood brain barrier in both directions, such that circulating BDNF might originate from neurons and glial cells of the brain. Thus, it has been suggested that changes in blood levels of BDNF are concomitant to its levels in brain [9].

There is evidence that development and maintenance of neural connections are disrupted in major central nervous system diseases, indicating a possible involvement of trophic factors in the pathogenesis of these disorders [10]. In this sense, abnormal BDNF signaling has been proposed to have a crucial role in the course and development of numerous psychiatric and neurological disorders [8]. Moreover, psychotropic drugs used to treat some of these conditions are known to activate BDNF signaling [8]. Extensive reviews have elaborated on the role of BDNF in individual disorders. In contrast, the goal of the present review is to give a more generalized overview of the involvement of BDNF in the pathology of the most prevalent psychiatric and neurological disorders, compiling what is known from human, animal, and in vitro studies.

## 2. Alzheimer’s Disease (AD)

AD is a complex progressive neurodegenerative disorder that results in memory deterioration and cognitive impairment [11]. The presence of β-amyloid (A-beta) peptide aggregates and neurofibrillary tangles (NFTs) composed of hyperphosphorylated tau protein are part of the neuropathological hallmarks of this disease [12]. A significant downregulation of BDNF messenger RNA (mRNA) and protein in AD has been strongly implicated in the loss of memory and cognitive function decline [12]. Specifically, three of seven transcripts of the human BDNF gene are under-expressed in AD [13]. While some studies may appear to describe conflicting results regarding decreases in BDNF signaling in AD, these discrepancies could be explained by the degree of the pathology, in that early-stage impairments do not necessarily correlate with a long-lasting and more severe condition. Postmortem reports revealed that in subjects with early AD, BDNF expression was not altered while TrkB expression was increased in hippocampus [14], whereas both BDNF and TrkB levels were reduced in cerebral cortex and hippocampus of patients with a severe AD pathology [15]. Buchman and colleagues reported the association between slower rates of cognitive decline with higher levels of BDNF expression in AD individuals [16]. Likewise, both pro-BDNF and m-BDNF protein levels have been found decreased in hippocampus and parietal cortex of AD brains [13], with reports of a 60% decrease in pro-BDNF hippocampal expression in AD patients compared to age-matched controls [17]. Reduced BDNF was also observed in the cerebrospinal fluid of AD patients [18].

Furthermore, BDNF polymorphisms have been associated with an increased risk of developing late-onset AD but the exact mechanisms remain unclear [12]. Particularly, a single nucleotide polymorphism within the BDNF gene causes a valine (Val) to methionine (Met) substitution at codon 66 (Val66Met) of the prodomain region. Carriage of 1 or 2 Met alleles is associated with lower BDNF production, decreased hippocampal volume, and cognitive decline [19]. In a middle-aged cohort with AD risk, carriage of the BDNF Met allele was associated with steeper decline in episodic memory and executive function, which in turn was exacerbated by a greater A-beta load. These results suggest that the BDNF Val66Met polymorphism could be considered as a target for novel AD therapeutics [19].

Transgenic mouse models of AD with A-beta overproduction have shown decreased cortical BDNF expression [12, 20] and a correlation between levels of large A-beta oligomers with a more drastic BDNF decrease [20]. Similarly, A-beta has been shown to downregulate BDNF levels in vitro [21]. Consistently, other transgenic mouse models of AD involving human tau expression with NFTs have shown a downregulation of BDNF mRNA [12]. These findings suggest that the two hallmarks of AD pathology, A-beta and NFTs, are linked to BDNF decreases.

Recently, DYRK1A, a serine threonine kinase with multiple targets, has attracted interest as a candidate AD biomarker [22]. Besides being involved in the control of excitation/inhibition balance, anti-inflammatory processes, and over-phosphorylation of tau protein, DYRK1A is also associated with the dysregulation of neurotrophic pathways, particularly at the level of BDNF. It has been proposed that a combined assessment in blood of DYRK1A along with BDNF and homocysteine has almost unequivocal rates of sensitivity, specificity and accuracy in testing for AD [22].

Among other major genetic risk factors for sporadic or late onset AD is apolipoprotein E4 (ApoE4) and mice expressing ApoE4 also have reduced BDNF levels [17]. It has been documented that ApoE isoforms differentially regulate maturation and secretion of BDNF from primary human astrocytes, with ApoE4 mediating a negative regulation of BDNF that could be lead to AD pathophysiology [17].

## 3. Parkinson’s Disease (PD)

PD is the second most common neurodegenerative disease after AD [11] and is characterized by the degeneration of dopaminergic neurons in the substantia nigra pars compacta (SNpc) and the subsequent reduction of dopamine in the striatum [23]. Its clinical manifestations include motoric (resting tremor, rigidity, bradykinesia and postural instability) and non-motor (olfactory deficits, constipation, sleep behavior disorders, cognitive impairment and mood disturbances, including anxiety and depression) symptoms [23]. At the cellular and molecular levels, PD is characterized by the appearance of Lewy bodies, which are composed of aggregated α-synuclein proteins [13]. PD etiology shows that the majority of cases are idiopathic or late onset (> 85 %), with a smaller proportion of familial and a high PD risk (< 10 %) [11].

BDNF is necessary for the establishment of dopaminergic neurons in the SNpc, and 70% of these neurons express BDNF and TrkB [24]. Besides the SNpc, BDNF is widely expressed in other brain structures critical for motor function, including the basal ganglia, cerebellum and brainstem [11]. In a bidirectional regulation, BDNF mediates the expression of dopamine receptors and tyrosine hydroxylase, the rate limiting enzyme for dopamine synthesis [11]. In animal models of PD, BDNF protects dopaminergic neurons against neurotoxin-induced neuronal death and failure or decrease in trophic support by this neurotrophin has been suggested to be an etiologic factor for PD [24]. Moreover, α-synuclein transcriptionally downregulates the expression of BDNF and alters its axonal trafficking [25]. These transport disturbances induced by α-synuclein have been deemed a key pathological factor in the progression of the disease [13]. Furthermore, BDNF has also garnered attention as a molecule crucial for cognition, a function that is compromised in PD patients [26].

Postmortem studies of PD patients found a reduction in the BDNF expression in the SNpc, caudate nucleus and putamen and these decreases in BDNF levels correlate to the degree of dopaminergic degeneration [11].

Other studies have investigated the relationship between the development of PD and the presence of a common polymorphism in the BDNF gene, Val66Met [24]. Val66Met affects cognitive and motor processes. This polymorphism decreases the interaction of pro-BDNF with sortilin (which is a cargo protein mediating the subcellular transport of pro-BDNF) and leads to decrease in BDNF dendritic distribution, reduced BDNF transport to secretory granules, and impairment of the activity-dependent secretory pathway of BDNF [24]. While Val66Met was associated with PD in a Japanese population, other studies found no association or considered it only a PD risk factor in European populations. Moreover, data from a Brazilian population indicated that Val66Met confers significant protection against PD and demonstrated that the amount of BDNF in the secretory pathway is diminished by the presence of at least one copy of the modified allele, although total amount of BDNF in the brain was not altered [24].

## 4. Huntington’s Disease (HD)

HD is a neurodegenerative disease caused by an extended polyglutamine tract in the huntingtin protein. The expression of mutant huntingtin protein (mHTT) is associated with ubiquitous molecular and cellular anomalies, widespread neuronal dysfunction and cell loss [27]. A hallmark of HD is the early degeneration of medium spiny neurons (MSNs) in the striatum, which constitute >95 % of its neuronal population [28]. As with AD and PD, HD presents motor and non-motor features, including progressive impairments in movement control, cognitive function and mood [27].

Striatal MSNs express TrkB but do not produce BDNF and thus they are critically dependent on their afferent supply of BDNF for neurotrophic maintenance [28]. Deficits in BDNF have been documented in cell lines expressing mHTT, in brains of HD mouse models, and in the caudate and putamen of HD patients posthumously, suggesting that volume loss in these regions may be mediated by a lack of neurotrophic support [27]. These deficits of neurotrophic sustenance caused by reduced levels of BDNF have been implicated in the selective vulnerability of striatal neurons in HD [6]. Indeed, reduced striatal TrkB expression has also been reported in knockin HD cellular and mouse models, exon-1 HD transgenic mice and HD human brain [6].

Impairment of the BDNF-TrkB pathway is suspected to underlie the early dysfunction and degeneration of striatal neurons in this disease. Studies with the transgenic R6/2 mouse model of HD found that prior to striatal degeneration there are early deficits in striatal levels of activated phospho-TrkB, while total-TrkB and BDNF levels remain unchanged [28]. These findings suggest that neurotrophic support of striatal neurons in HD is impaired early in disease progression due to defects in TrkB signal transduction [28].

Recent studies suggest that both receptors for BDNF, TrkB and p75NTR, are improperly regulated in the striata of HD patients and mouse models of HD [29]. While TrkB signaling almost exclusively promotes survival, p75 signaling may induce survival or apoptosis depending on the available ligand and associated co-receptor [29]. A reduction in TrkB mRNA expression has been reported in the caudate but not in the cortex of HD patients whereas the expression of p75NTR was increased [30]. This indicates that the neurotrophic deficits observed in HD could account not only for BDNF reduction but also for an imbalance between TrkB and p75NTR-mediated cell signaling. Evidence from HD transgenic mice shows that p75 signaling plays an early role in augmenting pro-survival effects in the striatum and that disruption of this signaling at a pre-symptomatic age may exacerbate the neuropathology [29]. Altogether, these findings support the idea that a balance between TrkB and p75NTR protein levels or their functional signaling cross-talk may be a factor to consider in the design of neuroprotective therapies in HD [6].

In addition to the decline in BDNF levels reported in the brains of patients with HD, it has been observed that administration of BDNF in HD mice protects against this disease’s neuropathology [31]. Although BDNF is produced in neurons, astrocytes are also an important source of this neurotrophin in the brain. Evidence has shown that mHTT decreases BDNF secretion from astrocytes by impairing the docking of BDNF vesicles on plasma membranes and thus affecting its exocytosis. This compromised exocytosis of BDNF in astrocytes in HD contributes to the decreased BDNF levels in affected brains and underscores the importance of improving glial function in the treatment of HD [31].

## 5. Depression

Depression is a serious and debilitating psychiatric disorder that may occur as early as 3 years of age and carries a high risk of suicide [32]. However, the precise neurobiology underlying this disorder is currently unknown. It has been proposed that an impairment of synaptic plasticity (neurogenesis, axon branching, dendritogenesis and synaptogenesis) in brain areas such as the hippocampus, may be a central element in the pathophysiology of depression and that these neural plasticity alterations may be related to abnormal levels of neurotrophic factors, specifically BDNF [33]. The hippocampus is smaller in depressed patients, but it is not clear whether reduced size is a consequence of depression or a pre-existing condition. The neurotrophic hypothesis of depression suggests that stress increases the susceptibility to depressive illness via increased hypothalamic–pituitary–adrenal axis activation, exposing the brain to corticosteroids and in turn, decreasing neurotrophic factors that are required for neuronal survival and function [34]. A recent review reported decreased BDNF expression in the hippocampus of animals under chronic stress conditions (modeling depression), and suggested that downregulation of BDNF seems to be associated with an increase in anxiety-like symptoms [10].

Post-mortem data show that levels of BDNF mRNA, circulating protein and full length TrkB, were significantly reduced in both hippocampus and prefrontal cortex of suicidal individuals compared with healthy controls [35, 36]. Along with the BDNF decreases in specific brain structures found in depressed individuals, levels of this neurotrophic factor have also been found to be reduced in blood [4, 37]. A comparison between depressed patients who attempted suicide, patients depressed but not suicidal and healthy controls, found significantly lower plasma BDNF levels in depressed suicidal patients compared to both other groups [9, 38]. Other reports using jugular vein blood sampling to directly correlate this venoarterial flow to brain levels of BDNF found that the venoarterial BDNF concentration was significantly reduced in patients at medium to high suicide risk [34]. Although some studies have not found differences in BDNF levels when comparing suicidal to non-suicidal individuals, it is clear that both depressed groups exhibit lower serum BDNF levels compared to healthy controls [39]. Similarly, BDNF expression was reduced in brains from adolescents who committed suicide [40]. Moreover, a polymorphism of the p75 receptor, which replaces a serine with a leucine residue, has been associated with attempted suicide in young adults with childhood-onset mood disorder [35]. The expression ratio of p75NTR to Trk receptors was found increased in the post-mortem brain of suicide subjects [41]. Reduced expression of BDNF, along with decreased Trk signaling and increased p75NTR, may indicate a shift from cell survival that could be associated with structural abnormalities and reduced neuronal plasticity in the suicidal brain [39].

Furthermore, a reduction in BDNF plasma levels was accompanied by a reduction in enzymes in charge of cleavage of pro-BDNF into m-BDNF in late-onset geriatric depression [42]. Decreased serum levels of m-BDNF in depressed patients have been more consistently reported whereas changes in pro-BDNF levels are less conclusive [4]. Karege and colleagues found a reduction in BDNF in suicidal individuals with the important additional observation that suicidal individuals receiving antidepressants had normal levels of BDNF [36]. Likewise, postmortem studies of the hippocampus of patients receiving antidepressant treatments revealed a higher BDNF immunoreactivity compared to non-treated individuals [43]. It has been proposed that antidepressants upregulate the adult hippocampal neurogenesis to treat depression [32]. Clinical response to these medications occurs following weeks to months of treatment suggesting that other phenomena than acute effects on monoaminergic systems is needed for antidepressants efficacy [33]. Indeed, antidepressant treatment increases BDNF levels, stimulates neurogenesis and reverses the inhibitory effects of stress [33], while infusions of BDNF into the hippocampus have antidepressant properties in rodent models of depression [44]. Lentiviral knockdown of BDNF in the hippocampus has been shown to reduce neurogenesis and precipitate behaviors associated with depression [44]. Furthermore, the ablation of hippocampal neurogenesis blocks the behavioral effects of antidepressants in animals [33].

## 6. Abuse Disorders

Cocaine addiction is a prevalent neuropsychiatric disorder that imposes especially high societal costs owing to its chronic relapsing nature [45]. Evidence also suggests a role for BDNF in mediating critical features underlying cocaine use disorder [46]. Cocaine self-administration results in a transient period of dephosphorylation of several proteins associated with neuronal plasticity. Reversing these proteins dephosphorylation with a single BDNF infusion into the prelimbic cortex immediately after a final session of cocaine results in suppression of relapse to cocaine-seeking for up to 4 weeks post infusion [45].

BDNF also appears to play a crucial role in the reward response to drugs such as heroin [47] and is involved in neuronal adaptation during opiate dependence [48]. BDNF serum levels were significantly lower in heroin-dependent patients at baseline compared to healthy controls [49], and were also decreased in patients undergoing different opiate-based maintenance treatments [47]. In addition, serum BDNF levels in these individuals showed a negative correlation with BDNF methylation rate [47]. BDNF expression is dramatically changed during opiate withdrawal, and is associated with drug withdrawal syndrome [49]. In animals, it was observed that morphine withdrawal was accompanied by upregulation of BDNF and TrkB, on the mRNA level in the frontal cortex, and only BDNF in hippocampus and midbrain [48].

Interestingly, alterations in BDNF signaling have also been linked to alcohol abuse disorders [7]. Alcohol abuse is a serious health problem worldwide that causes a variety of physical and mental disorders. Research has shown that BDNF plays an important role in alcohol addiction [50].

Studies in mice showed that chronic alcohol exposure upregulated BDNF and TrkB mRNA levels in the prefrontal cortex, but decreased sortilin and P75NTR mRNA levels in the dorsal striatum. Hence, chronic alcohol exposure induced a region-specific expression of BDNF and pro-BDNF and their respective receptors in the brain. These results suggest that BDNF and pro-BDNF signaling pathways may play major roles in alcohol preference and addiction [50]. In humans, serum BDNF levels are negatively correlated with the withdrawal severity in alcohol-ependent subjects [7]. In addition, DNA methylation of a BDNF promotor is increased in the serum of alcoholics compared to healthy controls and decreased during alcohol withdrawal [51]. Moreover, the Val-66Met polymorphism in humans has been associated with earlier onset of alcohol addiction, increased alcohol consumption, greater propensity to relapse, reduced gray matter during abstinence, and adverse alcohol abuse phenotypes in adolescents [7]. Mice carriers of this polymorphism exhibited a compulsive alcohol intake phenotype [52]. Furthermore, studies in rodents demonstrated that excessive alcohol consumption changes BDNF signaling by recruiting p75NTR in the dorsolateral striatum. In this line, it was shown that moderate consumption of alcohol increases BDNF expression in the dorsolateral striatum through activation of TrkB. In contrast, knockdown of the BDNF gene increases the intake of moderate amounts of alcohol in mice and rats [7]. As a corollary, it appears that BDNF plays a pivotal role in the mechanisms governing alcohol abuse and suggests that modulators of BDNF signaling could be developed as treatments to prevent a pathological use of alcohol [7].

## 7. Schizophrenia

Schizophrenia is one of the most common psychiatric disorders worldwide resulting from a complex interplay between genetic and environmental risk factors [53, 54]. Suicide is a common occurrence in schizophrenia with 25%–30% patients attempting suicide and 8%–10% completing it [53]. A growing number of studies implicate BDNF in the pathogenesis of schizophrenia [54, 55], and consider it an important element in the protection of neurons and in the prevention of the neurobiological changes that may lead to suicide in this disorder [56].

In addition, mRNA levels of BDNF and TrkB have been found to be reduced in the prefrontal cortex and hippocampus of patients with schizophrenia [53]. Along with these changes, functional abnormalities have been observed in the hippocampus of schizophrenic individuals, who exhibit a significant reduction in hippocampal volume [55]. A longitudinal study using imaging measures found that serum BDNF levels were associated with hippocampal volume alterations in schizophrenia patients [55]. There seems to be a consensus about the peripheral BDNF levels being decreased in schizophrenia. Several studies report reduced blood levels of BDNF in both medicated and drug-naïve patients [54, 57-59]. In contrast, the effects of treatment with antipsychotics or other therapies on BDNF levels seem to be controversial. While some reports of treatment with atypical antipsychotics [60] and a combination of electroconvulsive therapy with antipsychotics [58] have found increases in serum BDNF levels, other studies particularly with antipsychotics in animals [57] and schizophrenic patients [54] have not found any effect on BDNF levels.

Carriers of the Val66Met polymorphism of BDNF reported decreased BDNF plasma levels in schizophrenia-affected individuals compared to healthy subjects [54].

## 8. Epilepsy

Temporal lobe epilepsy is the most common and often devastating form of human epilepsy. Although the molecular mechanisms underlying the development of this condition remain largely unknown, emerging evidence suggests that activation of the BDNF receptor TrkB promotes epileptogenesis [61]. In a mouse model of recurrent seizures, a transient inhibition of TrkB after status epilepticus and continued for 2 weeks was found to prevent recurrent seizures and limit the loss of hippocampal neurons [61]. Other studies in animals indicate that serum BDNF levels are predictive for the vulnerability to develop epilepsy after an insult, and for the development of comorbidities such as depression and cognitive deficits [62]. These findings are consistent with the reports of increased tissue levels of BDNF once epilepsy has developed [62]. Evidence from rodent studies suggests that pro-BDNF signaling through p75NTR may account for the emergence of epileptic disorders [63]. In rats, increased pro-BDNF/p75NTR signaling disrupted the excitation/inhibition balance and enhanced neuronal network activity, leading to an increase in seizure susceptibility that was abolished by blocking p75NTR [63].

## 9. Conclusion

Neurotrophic support mediated by BDNF is a critical factor for the development and maintenance of neural connections. Compelling evidence has linked the pathogenesis of several major neurologic and psychiatric disorders to alterations in BDNF function. Hence, design of therapies and pharmacological interventions for treating these disorders should benefit from restoration of BDNF signaling.
